# Robust Attitude Estimation for Low-Dynamic Vehicles Based on MEMS-IMU and External Acceleration Compensation

**DOI:** 10.3390/s24144623

**Published:** 2024-07-17

**Authors:** Jiaxuan Chen, Bingbo Cui, Xinhua Wei, Yongyun Zhu, Zeyu Sun, Yufei Liu

**Affiliations:** 1 Key Laboratory of Modern Agricultural Equipment and Technology, Jiangsu University, Ministry of Education, Zhenjiang 212013, China; 2212116055@stmail.ujs.edu.cn (J.C.); wxh@ujs.edu.cn (X.W.); yyz@ujs.edu.cn (Y.Z.); 1000004203@ujs.edu.cn (Z.S.); 2 School of Agriculture Engineering, Jiangsu University, Zhenjiang 212013, China; 3 College of Biosystems Engineering and Food Science, Zhejiang University, Hangzhou 310058, China; yufeiliu@zju.edu.cn

**Keywords:** dynamic attitude estimation, inertial measurement unit, robust Kalman filter, external acceleration compensation

## Abstract

Attitude determination based on a micro-electro-mechanical system inertial measurement unit (MEMS-IMU) has attracted extensive attention. The non-gravitational components of the MEMS-IMU have a significant effect on the accuracy of attitude estimation. To improve the attitude estimation of low-dynamic vehicles under uneven soil conditions or vibrations, a robust Kalman filter (RKF) was developed and tested in this paper, where the noise covariance was adaptively changed to compensate for the external acceleration of the vehicle. The state model for MEMS-IMU attitude estimation was initially constructed using a simplified direction cosine matrix. Subsequently, the variance of unmodeled external acceleration was estimated online based on filtering innovations of different window lengths, where the acceleration disturbance was addressed by tradeoffs in time-delay and prescribed computation cost. The effectiveness of the RKF was validated through experiments using a three-axis turntable, an automatic vehicle, and a tractor tillage test. The turntable experiment demonstrated that the angle result of the RKF was 0.051° in terms of root mean square error (RMSE), showing improvements of 65.5% and 29.2% over a conventional KF and MTi-300, respectively. The dynamic attitude estimation of the automatic vehicle showed that the RKF achieves smoother pitch angles than the KF when the vehicle passes over speed bumps at different speeds; the RMSE of pitch was reduced from 0.875° to 0.460° and presented a similar attitude trend to the MTi-300. The tractor tillage test indicated that the RMSE of plough pitch was improved from 0.493° with the KF to 0.259° with the RKF, an enhancement of approximately 47.5%, illustrating the superiority of the RKF in suppressing the external acceleration disturbances of IMU-based attitude estimation.

## 1. Introduction

Accurate attitude information not only plays a crucial role in vehicle navigation and localization, but is also an important parameter for operational control of agricultural implements, such as monitoring and controlling tractor tillage depth [1,2,3]. Generally, the variation of vehicle attitude can be updated by integrating the output of gyroscope angular rates. However, lack of knowledge of the initial vehicle attitude and gyroscope bias may lead to drift in attitude calculation. This issue is particularly significant when dealing with micro-electro-mechanical system (MEMS) gyroscopes, as their noise characteristics and environmental drift errors are highly complicated. Based on the accelerometer’s specific force measurements, calculation of attitude angles can be achieved under static and uniform motion conditions of the vehicle. However, the measurements are affected by external accelerations [4,5,6]. Attitude estimation based on a low-cost inertial measurement unit (IMU) has attracted significant attention due to the complementary error characteristics of gyroscope and accelerometer in calculating the vehicle attitude [7,8,9].

Popular attitude estimation methods include complementary filter (CF) [10,11] and extended Kalman filter (EKF) [12,13]. The CF method utilizes frequency complementary features of the gyroscope-derived and accelerometer-derived attitude. The EKF method constructs a measurement model based on the difference between the accelerometer’s observations and the projection of gravitational effects on the vehicle body axis, and it updates the attitude in the time domain based on the gyroscope output. While the CF method is characterized by reduced computational complexity compared to the Kalman filter (KF), its rate of convergence is impacted by the initial attitude estimation [14]. Furthermore, the KF exhibits greater flexibility compared to the CF in model design, especially in the case of dynamic conditions. The KF method and its variants have found widespread applications in the field of autonomous driving [15,16,17,18,19]. With the increase of computational capabilities for microcontrollers, there is a growing advantage in exploring KF-based methods for attitude estimation in navigation and control applications [20,21,22]. Many robust KFs have been proposed for the measurement of outlier detection in GNSS-based navigation [23,24], which however cannot be applied in IMU-based attitude estimation due to the attitude modulation of accelerometer observations. Javed et al. proposed a cascaded KFs structure to compensate gyroscope biases and imported an adaptive external acceleration model to detect external acceleration [25]. Odry et al. utilized IF-THEN rule-based adaptation laws to adjust noise covariance matrices dynamically, which suppressed external disturbances effectively [26]. The above-mentioned KF-based attitude estimators primarily rely on adaptive measurement noise setting to modify the Kalman gains once external acceleration is detected. However, there is a distinct difference between external acceleration and accelerometer noise, which is that the modeling error of external acceleration is scenario-dependent and cannot be performed once for all conditions. Consequently, further investigation is required to improve the accuracy of attitude control under different dynamic conditions.

In the navigation of agricultural machinery and operational control of implements, KF-based attitude estimation has been widely employed to correct positioning errors or improve implement control stability [27,28]. Huang et al. developed a precise tilt angle monitor system by using the Euler angle algorithm to calculate attitude, where the noise covariances were adjusted adaptively to handle time-varying working conditions [29]. Yu et al. improved the implement leveling control system by reducing the implement attitude estimation error based on combined information from gyroscope and accelerometer [30]. Yang et al. proposed a pitch angle prediction model for tractors based on time series analysis and an EKF, which improved the dynamic response speed of tilling depth regulation [31]. Zhao et al. proposed an improved adaptive Kalman filtering method for sowing depth detection after analyzing the movement mechanism of a seed drill’s parallel four-bar linkage [32]. All of these attitude prediction model-based control systems share a common premise: that the attitude estimation model can address the actual attitude of the agricultural implement, and external accelerations are neglected. However, in the case of frequent vibration and uneven soil conditions for heavy machinery, such as in the design of tractor tillage depth control systems, this premise is not true and the external acceleration may negatively affect the attitude estimation.

Recently, Candan et al. proposed an adaptive external acceleration compensation method based on KF innovations, where a diagonal weight matrix was designed for triaxial accelerometers, and the method was verified using IMU data from an unmanned aerial vehicle (UAV) [33]. In this paper, we further validate the robust KF (RKF) for attitude estimation of low-dynamic vehicles, such as an automatic control vehicle and tractor, where the window length selection for attitude estimation under different dynamic conditions is investigated. Experiments based on turntable tests, automatic control vehicles and tractor tillage field tests were employed to evaluate the performance of the RKF. The experimental results can provide a valuable reference on how to compensate for external acceleration in attitude determination of a low-dynamic vehicle.

The structure of this paper is arranged as follows. The simplified attitude filter model is briefly reviewed in Section 2, and then the robust KF-based attitude estimation is presented. In Section 3, the RKF is verified by employing a turntable experiment, automatic vehicle and tractor field test. Finally, Section 4 concludes this work.

## 2. Materials and Methods

### 2.1. KF Algorithm

The KF algorithm consists of time-updating and measurement-updating stages. The discrete state-space model (SSM) can be written as follows:

(1) $$x_{k}=\Phi_{k-1}x_{k-1}+w_{k-1}$$

(2) $$z_{k}=H_{k}x_{k}+v_{k}$$

where $x_{k}$ is the system state at time *k*; $\Phi_{k-1}$ is the system state transition matrix; $z_{k}$ is the system measurement vector; $H_{k}$ is the system measurement matrix; while $w_{k-1}$ and $v_{k}$ are the system noise and measurement noise, respectively; the initial state satisfies $N\left(x_{0},P_{0}\right)$ which is independent with $w_{k-1}$ and $v_{k}$, where $N\left(x_{0},P_{0}\right)$ denotes a Gaussian distribution with mean $x_{0}$ and variance $P_{0}$. The KF time update is formulated as [15]

(3) $$x_{k}^{-}=\Phi_{k-1}x_{k-1}$$

(4) $$P_{k}^{-}=\Phi_{k-1}P_{k-1}\Phi_{k-1}^{T}+Q_{k-1}$$

where $x_{k}^{-}$, $P_{k}^{-}$ are the prediction state and corresponding covariance matrix, and $Q_{k-1}$ is the system noise covariance matrix. The measurement update of the KF can be written as

(5) $$K_{k}=P_{k}^{-}H_{k}^{T}{\left(H_{k}P_{k}^{-}H_{k}^{T}+R_{k}\right)}^{-1}$$

(6) $$x_{k}=x_{k}^{-}+K_{k}e_{k}$$

(7) $$P_{k}=\left(I-K_{k}H_{k}\right)P_{k}^{-}$$

where $K_{k}$ is KF gain; $e_{k}=z_{k}-H_{k}x_{k}^{-}$ is the filtered innovation; I is the identity matrix of proportional dimension; and $R_{k}=E\left[v_{k}v_{k}^{T}\right]$ is the noise covariance matrix. The KF operates under the assumption that the state estimation at time *k* depends solely on the state at the previous time and the measurement value at the current time. Through iterative processes, it estimates the state and its uncertainty in the temporal domain, achieving optimal estimation when Gaussian noise is assumed in the linear state space.

### 2.2. IMU Attitude Estimation

#### 2.2.1. Principle of Attitude Calculation

The angular rate and specific force of the vehicle frame (b-frame) relative to the inertial frame (i-frame) are measured by the gyroscopes and accelerometers of the IMU. Calculating the vehicle’s attitude angle under dynamic conditions involves solving the attitude differential equations using the outputs of the gyroscopes and transforming them into the navigation frame (n-frame). In this work, the navigation frame is selected as North-East-Down, and the vehicle frame is set as Front-Right-Down. Popular attitude updating methods include direction cosine matrix (DCM) and quaternion updating. In this study, a simplified DCM is employed to determine the vehicle’s roll and pitch angles. The transformation matrix from b-frame to n-frame can be formulated as

(8) $$C_{b}^{n}=\left[\begin{array}{ccc} c\alpha c\beta & c\alpha s\beta s\gamma -s\alpha c\gamma & c\alpha s\beta c\gamma +s\alpha s\gamma \\ s\alpha c\beta & s\alpha s\beta s\gamma +c\alpha c\gamma & c\alpha s\beta s\gamma -c\alpha s\gamma \\ -s\beta & c\beta s\gamma & c\beta c\gamma \end{array}\right]$$

where *s* denotes the sine function; *c* denotes the cosine function; *α*, *β*, and *γ* are the rotation angles of the b-frame around the Z, Y, and X axis, respectively. Consequently, the pitch and roll angles are defined as follows:

(9) $$\beta =\tan^{-1}\left(\frac{-C_{31}}{\sqrt{{C_{33}}^{2}+{C_{32}}^{2}}}\right)$$

(10) $$\gamma =\tan^{-1}\left(\frac{C_{32}}{C_{33}}\right)$$

where $C_{ij}$ is the element of matrix $C_{b}^{n}$ at the *i*th row and *j*th column. Due to the existence of gravity, based on Equation (8) the projection of gravity to b-frame gravity can be expressed as follows:

(11) $$g^{b}={\left(C_{b}^{n}\right)}^{T}g^{n}=\left(\begin{array}{c} -s\beta \\ c\beta s\gamma \\ c\beta c\gamma \end{array}\right)\cdot g_{e}$$

where $g_{e}$ is the local gravitational acceleration. In Equations (6)–(8), the last row of $C_{b}^{n}$ not only calculates *β* and *γ*, but can also be used to compensate for the effect of acceleration measurements $g_{e}$, thereby the state vector of attitude SSM is selected as

(12) $$x_{k}={\left(C_{31},C_{32},C_{33}\right)}^{T}$$

#### 2.2.2. Filtering Model Construction

The gyroscope and accelerometer measurements at time *k* can be expressed as follows:

(13) $$y_{g,k}=\omega_{ib,k}^{b}+n_{g,k}$$

(14) $$y_{a,k}=a_{k}+g^{b}+n_{a,k}$$

where $y_{g,k}$ and $y_{a,k}$ are the outputs of the gyroscope and accelerometer; $\omega_{ib,k}^{b}$ and $a_{k}$ are the ideal angular rate and external acceleration; while $n_{g,k}$ and $n_{a,k}$ are the zero-mean Gaussian white noise of the sensor. Suppose the biases of gyroscope and accelerometer have been compensated and we can thus ignore the random bias error in (13) and (14). The external acceleration is assumed to change slowly and can be modeled using a first-order low-pass filtered white noise process.

(15) $$a_{k}=c_{a}a_{k-1}+\varepsilon_{k}$$

where $c_{a}$ is a constant ranging from 0 to 1 and $\varepsilon_{k}$ is the time-varying error in modeling the vehicle’s acceleration motion. The first-order approximation is employed to solve the differential equation of DCM at time *k*, then we have

(16) $$C_{b,k}^{n}=C_{b,k-1}^{n}\left(I_{3}+∆T\left(\omega_{ib,k}^{b}\times \right)\right)$$

where $\omega_{ib,k}^{b}$ is the ideal measurement of the gyroscope; $\left(\omega \times \right)$ denotes the skew-symmetric matrix of the vector ω, and ∆T is the sampling period of the gyroscope. It is notable that the earth rate $\omega_{ie,k}^{b}$ is neglected in deriving (16), as the gyroscope may not be sufficiently accurate to distinguish earth rate from random noise. Substituting the actual measurement from Equation (13) into the above equation and corresponding to Equation (1) yields the following results:

(17) $$\Phi_{k-1}=I_{3}-∆T\left(y_{g,k}\times \right)$$

(18) $$w_{k-1}=∆T\left(-x_{k-1}\times \right)n_{g,k}$$

Subsequently, the system noise covariance matrix is expressed as

(19) $$Q_{k-1}=-∆T^{2}\left(x_{k-1}\times \right)\Sigma_{gg}\left(x_{k-1}\times \right)$$

where $\Sigma_{gg}$ is the noise covariance of the gyroscope measurement. By substituting Equation (15) into Equation (14) and associating it with Equation (11), we have

(20) $$y_{a,k}-c_{a}a_{k-1}=g_{e}x_{k}+\varepsilon_{k}+n_{a,k}$$

Corresponding to Equation (2), the following expressions can be obtained:

(21) $$z_{k}=y_{a,k}-c_{a}a_{k-1}$$

(22) $$H_{k}=g_{e}I_{3}$$

(23) $$v_{k}=\varepsilon_{k}+n_{a,k}$$

Since $\varepsilon_{k}$ and $n_{a,k}$ are uncorrelated noise terms, we have

(24) $$R_{k}=\Sigma_{acc,k}+\Sigma_{aa}$$

where $\Sigma_{aa}$ is the accelerometer noise covariance and $\Sigma_{acc,k}$ is the corresponding variance of the external acceleration modeling error. Based on Equations (17)–(24), a simplified DCM attitude estimation model can be derived. In this model, the external acceleration motion modeling error of the mobile vehicle is addressed by $R_{k}$, which affects the attitude estimation results and must be suppressed during dynamic attitude estimation of the vehicle.

### 2.3. Robust Attitude Estimation Method

Attitude estimation based on adaptive measurement noise has been applied in UAVs [33], whereas its performance in processing vibration for low-dynamic vehicles is seldom reported. This study focuses on measurement anomaly detection and an online noise estimation approach based on KF innovation. It also aims to compensate for measurement model uncertainty and assess the impact of its application on attitude estimation in the presence of vibration interference on low-dynamic vehicles. The innovation of the KF denotes the deviation between predicted and actual measurements. It accurately reflects the deviation of the estimated state from the actual underlying state, with its covariance defined as the covariance between predicted and actual measurements. The innovation covariance of the KF is expressed as

(25) $$C_{k}=E\left[e_{k}e_{k}^{T}\right]{=H}_{k}P_{k}^{-}H_{k}^{T}+{\hat{\Sigma }}_{acc,k}+\Sigma_{aa}$$

Subsequently, Kalman gain matrix is determined as

(26) $$K_{k}=P_{k}^{-}H_{k}^{T}{\left(H_{k}P_{k}^{-}H_{k}^{T}+{\hat{\Sigma }}_{acc,k}+\Sigma_{aa}\right)}^{-1}$$

where ${\hat{\Sigma }}_{acc,k}$ is the online estimated value at moment *k*. When there is an external acceleration for the vehicle, ${\hat{\Sigma }}_{acc}$ increases, $K_{k}$ decreases, and $x_{k}$ is more dependent on the time update of the KF. To minimize the computational load of the RKF, the variance of the noise in the measurement associated with external acceleration is updated only when a substantial deviation exists between the trace value of detected and predicted innovation covariance, that is

(27) $$tr(e_{k}{e_{k}}^{T})>tr\left(H_{k}P_{k}^{-}H_{k}^{T}+{\hat{\Sigma }}_{acc,k}+\Sigma_{aa}\right)$$

where tr(·) is the operation of matrix trace calculation. It is assumed that in case the KF becomes stable, only $\Sigma_{acc,k}$ in the innovation covariance changes due to external acceleration. To detect anomalous measurements at time *k*, ${\hat{C}}_{k}$ can be approximated by averaging the values of $C_{k}$ over multiple instances of the filtering update, which can be estimated in real-time by

(28) $${\hat{C}}_{k}=\frac{1}{\mu }\sum_{j=k-\mu +1}^{k}e_{j}{e_{j}}^{T}$$

where *μ* is the time window length. In order to estimate ${\hat{\Sigma }}_{acc}$, we set

(29) $$\frac{1}{\mu }\sum_{j=k-\mu +1}^{k}e_{j}{e_{j}}^{T}=H_{k}P_{k}^{-}H_{k}^{T}+ST+\Sigma_{aa}$$

where ST is the matrix variable corresponding to the RKF, then we have

(30) $$ST=\frac{1}{\mu }\sum_{j=k-\mu +1}^{k}e_{j}{e_{j}}^{T}-H_{k}P_{k}^{-}H_{k}^{T}-\Sigma_{aa}$$

When the condition of Equation (27) is satisfied, the RKF uses Equation (30) to update the adjustment matrix ST. Due to the limited correlation among the effects of external acceleration to the vehicle in the three axial directions of the accelerometer, ${\hat{\Sigma }}_{acc,k}$ is updated as follows:

(31) $${\hat{\Sigma }}_{acc,k}=diag\left(s_{1},s_{2},s_{3}\right)$$

(32) $$s_{i}=max\left(0,\;S_{ii}\right)$$

where $S_{ii}$ is the *i*th diagonal element of ST, *i* = 1, 2, 3, and we set $s_{i}=max\left(0,\;S_{ii}\right)$ to denote the external acceleration modeling error always larger than 0.

**Figure 1:**
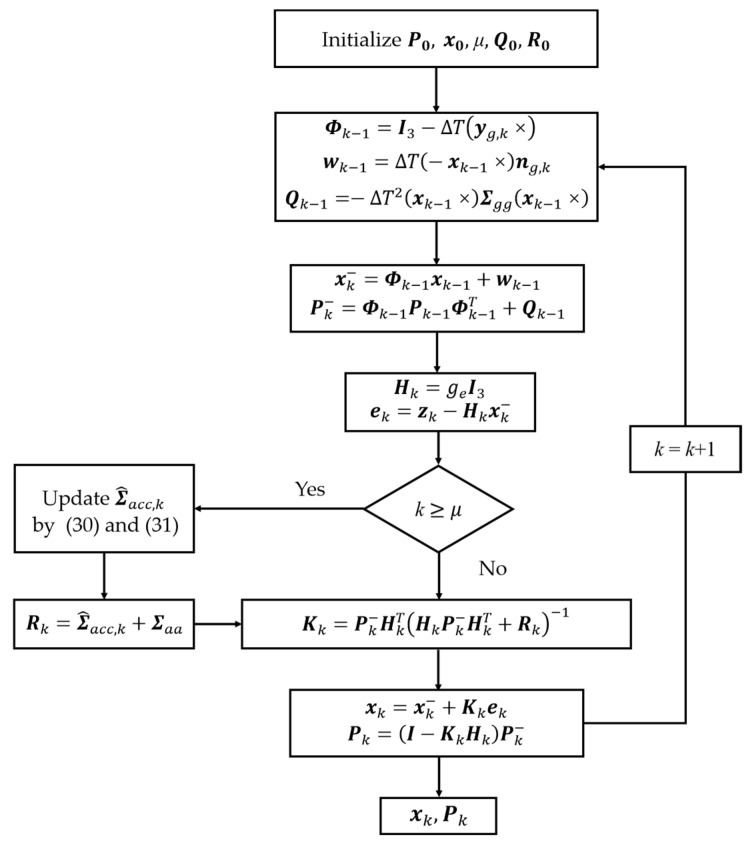
Flowchart of attitude estimation based on RKF.

The flowchart of attitude estimation based on multi-scale disturbance compensation is illustrated in Figure 1. The time window length *μ* utilized in calculation ${\hat{C}}_{k}$ not only affects ${\hat{\Sigma }}_{acc,k}$ calculation results but also impacts the sensitivity of anomaly detection in measurements. The selection of *μ* is a tradeoff among prescribed computational cost, time-delay and dynamic accuracy of the attitude estimator. The subsequent experiments in this paper will further explore the effects of varying values of *μ* on attitude estimation for low-dynamic vehicles.

## 3. Results

### 3.1. Turntable Angle Tracking Test

**Figure 2:**
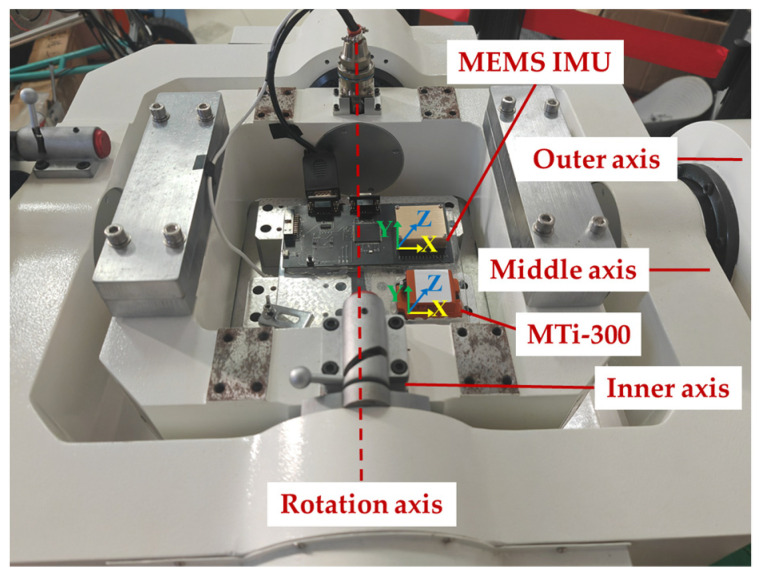
Turntable test.

The effectiveness of the RKF was evaluated through experimentation on a three-axis continuously rotating turntable, where its performance under static or quasi-static conditions was investigated. The turntable exhibited an inclination rotation error of ±5″, an angular positioning accuracy of ±4″, and a minimum angular rate of 0.001°/s. The attitude measurement module is self-designed based on a microcontroller unit (STM32F429) and MEMS-IMU (KY-IMU102N-A0), where the latter is provided by Beijing Beidou Satellite Communication Group Co., Ltd. (Beijing, China). The raw IMU data was transmitted to STM32F429 via a serial peripheral interface, where the constant biases of gyroscope and accelerometer were well compensated. The gyroscope exhibited a zero-bias stability of 12°/h (with 10 s smoothing), and its zero-bias repeatability was 0.24°/h. The zero-bias stability of the accelerometer was 60 µg, and its zero-bias repeatability was 1 mg. A widely applied attitude and heading reference system (AHRS), MTi-300, provided by Xsens Company (Enschede, The Netherlands), was utilized to assess the performance of the developed algorithm in monitoring the turntable’s angle changes. The MTi-300 consists of triaxial gyroscopes with zero-bias stability of 10°/h, and triaxial accelerometers with zero-bias stability of 40 µg, which are comparable to our self-designed attitude measurement board. The nominal attitude accuracy of the MTi-300 is 0.2° in root mean square. In Figure 2, the self-designed attitude measurement board and MTi-300 are affixed to the fixture and then fixed on the turntable. Initially, the inner axis of the turntable (corresponding to the Y-axis of the IMU and MTi-300) remained stationary and was subsequently rotated to the 10° position at an angular velocity of 5°/s and an angular acceleration of 10°/s^2^. The output data from the IMU and AHRS were concurrently recorded at a data sampling rate of 100 Hz. The turntable data was captured through external synchronized trigger acquisition, and all the attitude results were synchronized and saved simultaneously for post-analysis. To guarantee the comparability of the KF and RKF estimation methods, the same parameters were set for the two filters, with the exception that the RKF was set to *μ* = 1.

**Figure 3:**
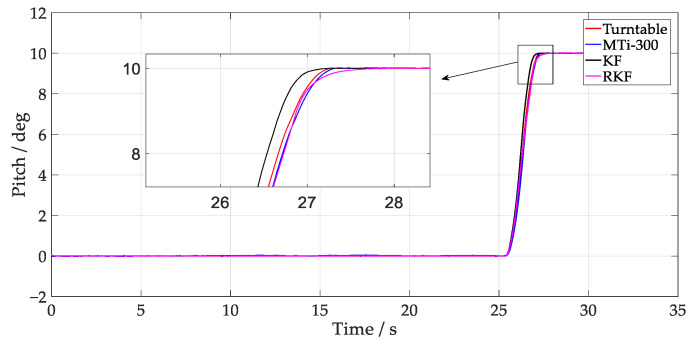
Results of pitch tracking test.

**Figure 4:**
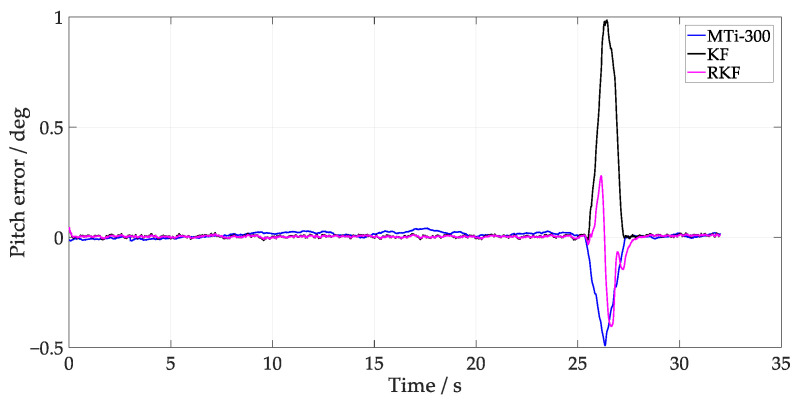
Pitch estimation of different methods.

The results depicting the estimation of the turntable test angles are presented in Figure 3 and Figure 4, where the MTi-300 and RKF demonstrate superior tracking of the turntable attitude change compared to the KF. Given that the gyroscope output angular rate impacts attitude estimation during rapid external angle changes (26~28 s), the attitude estimation was predominantly influenced by the gyroscope’s output angular rate. In KF time updates, an unmodeled error in $Q_{k-1}$ affects the consistency of the predicted covariance matrix estimation. It is noteworthy that during these angular changes, the RKF showed a zero-crossing error, which may result from the significant variation of innovation covariance. When the $R_{k}$ of the RKF is increased, the Kalman gain becomes small, making the RKF depend more on the integration of gyroscope outputs. Once the online computation of ST decreases $R_{k}$, the RKF depends more on accelerometer outputs, resulting in a changed estimation error in different directions. By taking the turntable output angle as the reference, Table 1 presents the root mean square error (RMSE) of different methods. The result indicates that the angle error of the RKF is 0.051°, demonstrating improvements of 65.5% and 29.2% compared to the KF and MTi-300, respectively.

### 3.2. Dynamic Attitude Estimation Test

**Figure 5:**
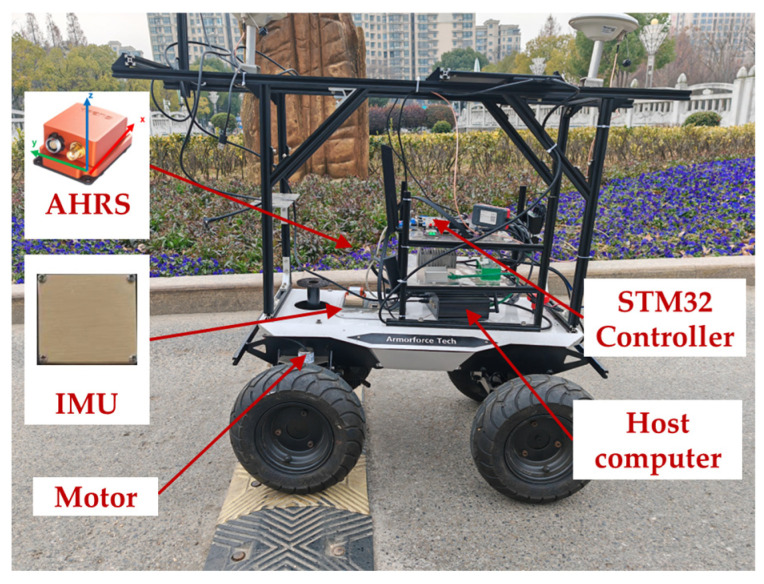
Attitude estimation test of the automatic vehicle.

Low-dynamic vehicles frequently suffer from angular or linear vibrations, which may degrade attitude estimation based on a MEMS-IMU without acceleration compensation. In order to further verify the performance of the RKF under the condition of external acceleration disturbance, a dynamic attitude test platform was built based on the automatic vehicle shown in Figure 5. The self-designed attitude measurement board and MTi-300 were fixed on the test vehicle, and the sampling frequency of the IMU and MTi-300 were set to 100 Hz. In the test, the automatic vehicle was controlled to pass through speed bumps at different speeds to simulate the interference of external vibration on the specific force observation of the accelerometer. To match the dynamic change process of the carrier, the RKF was set to *μ* = 1, and the automatic vehicle ran at an average speed of 0.5 m/s (low speed) and 1.5 m/s (medium speed), respectively.

**Figure 6:**
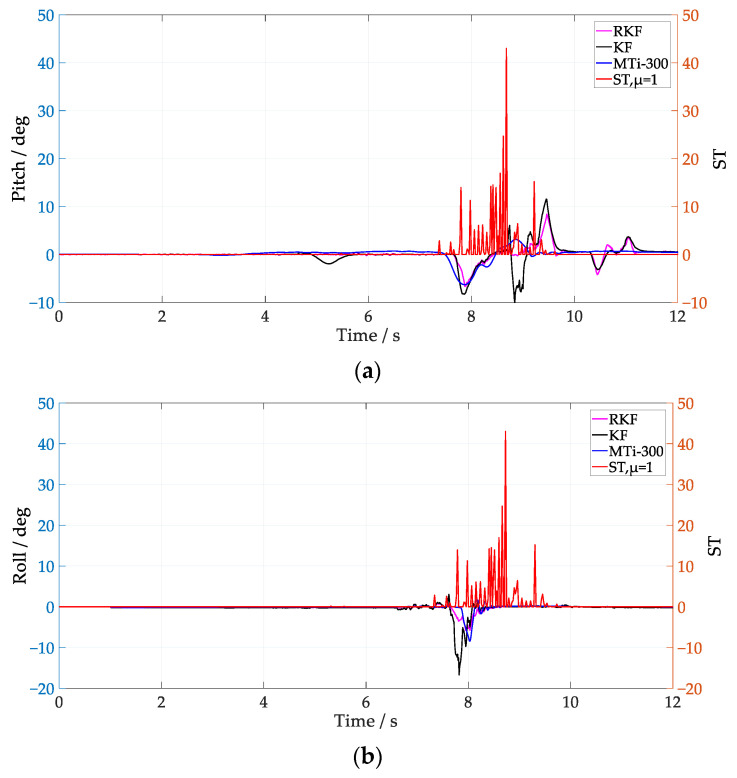
Attitude estimation at low speed with μ=1. (**a**) Pitch; (**b**) roll.

**Figure 7:**
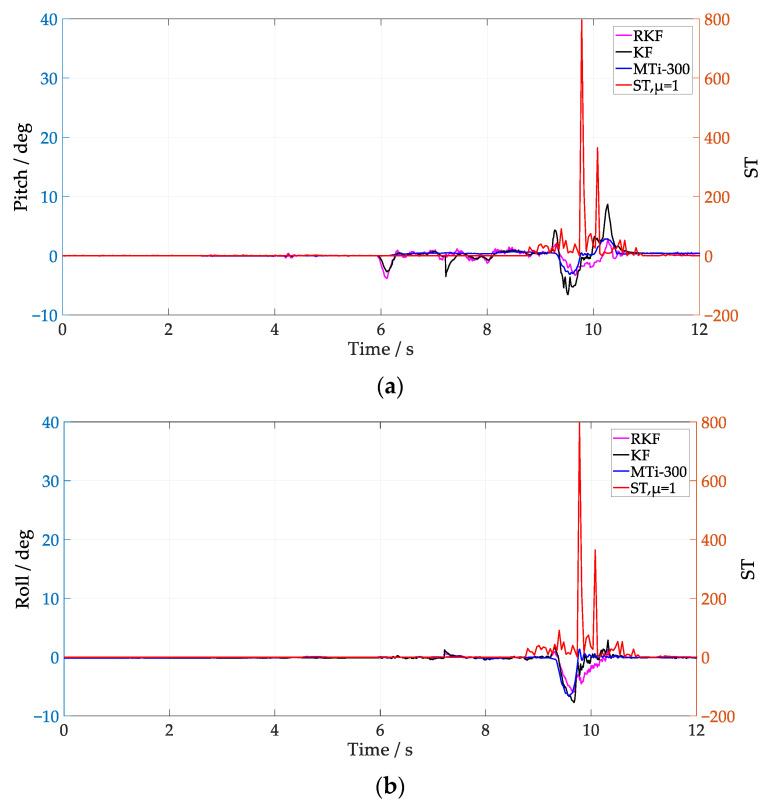
Attitude estimation at medium speed with μ=1. (**a**) Pitch; (**b**) roll.

The corresponding attitude results are shown in Figure 6 and Figure 7. Since the attitude estimation of the KF is affected by external acceleration interference, and its pitch has obvious abrupt changes, the RKF can effectively smooth the abrupt change of pitch by using innovation variance and external acceleration error compensation. Comparing the attitude estimation results for low speed and medium speed, it is evident that as the vehicle speed increases, even slight vibrations during the autonomous vehicle’s movement can significantly impact the attitude estimation results. Taking the attitude of the MTi-300 as the reference value, the attitude estimation error of the KF and the RKF are shown in Table 2. It is notable that the RKF suppresses the external acceleration interference better than the KF under different speed conditions, and its pitch and roll are improved by about 57% and 74.8% at low speed, and about 47.4% and 45.7% at medium speed.

**Figure 8:**
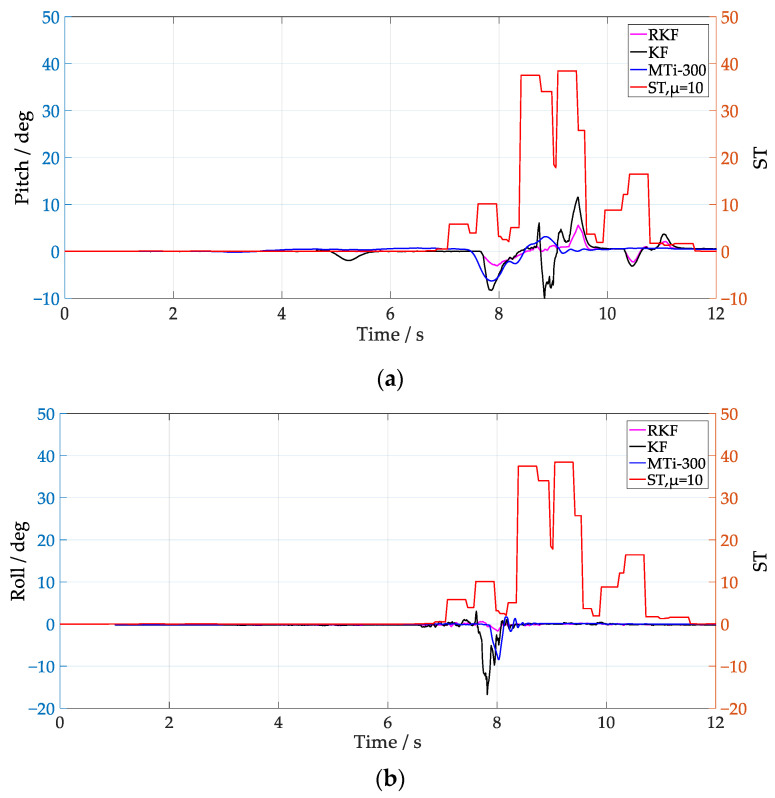
Attitude estimation at low speed with μ=10. (**a**) Pitch; (**b**) roll.

From Figure 6 and Figure 7, it can be seen that the ST values fluctuate significantly when the vehicle passes over speed bumps. In Figure 6, the RKF does not show significant improvement over the KF in the pitch between 10 s and 12 s, possibly because the condition of Equation (27) is not met, and the RKF does not compensate for the acceleration modeling error. Notice that, by employing *μ* = 1, the ST values at medium speed clearly increase compared with that of the low-speed scenario, which coincides with the fact that the external acceleration of the former scenario is larger. It is notable that the improvement of roll is better than pitch when the vehicle runs at low speed, while it is not the case when the vehicle runs at medium speed, i.e., both of them show similar improvement. The different performance in roll and pitch estimation indicate that the multi-scale acceleration compensation based on Equation (31) can decouple the external acceleration projected in different sensitive axes of the IMU. In order to further analyze the influence of Equation (27) on attitude anti-interference estimation, *μ* = 10 was selected to process the IMU data at low speed, and the results are shown in Figure 8. Notice that the RKF pitch estimation results are improved between 10 s and 12 s by increasing the time window length of the innovation estimation. By increasing the value of *μ*, the innovation variance can be estimated more accurately, and thus the sensitivity and precision of the external acceleration model are improved.

**Figure 9:**
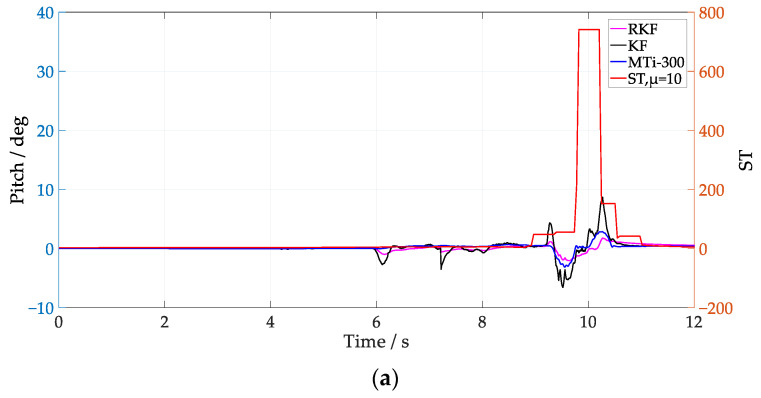

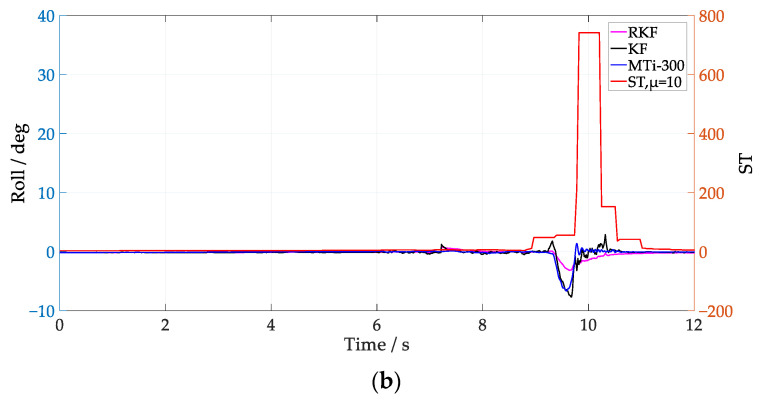
Attitude estimation at medium speed with μ=10. (**a**) Pitch; (**b**) roll.

The attitude estimation results with *μ* = 10 under medium speed conditions are shown in Figure 9, and compared with Figure 7, it can be seen that the pitch is significantly improved in the range of 6 s–8 s. When the ST value in the 9 s–11 s interval changes significantly, the pitch and roll of the RKF are smoother than those of the KF, and maintain a similar trend with that of the MTi-300. Comparing the ST values under different *μ* selections, it can be seen that increasing *μ* can obtain a more accurate covariance of the external acceleration modeling error, but its dynamic response ability to external interference is degraded, which is further verified by the slow convergence trend of the roll of the RKF between 10 s–12 s in Figure 9.

### 3.3. Plough Attitude Estimation Test

**Figure 10:**
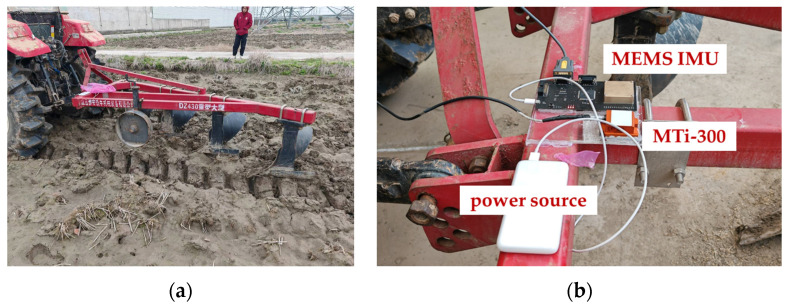
Attitude estimation of plough. (**a**) Setup for plough attitude estimation; (**b**) sensors installation diagram.

In this section, the result of plough attitude estimation is reported by employing a tractor tillage platform under actual field conditions, where the uneven soil conditions and soil moisture lead to frequent external acceleration. As shown in Figure 10, the platform is constructed using the Dongfanghong 1104 tractor, with a self-designed attitude measurement board and an MTi-300 mounted on the suspended ploughing tool. To compensate for external acceleration of different frequencies, the sampling rate of the IMU and the MTi-300 are set to 100 Hz. The tractor works at its standard ploughing operational speed. Vibration interference comes from both soil and mechanical system during the work process and has a significant effect on the estimation of pitch angle, leading to obvious differences in pitch compared with that of the MTi-300. The novel KF innovation variance estimation method is employed to compensate for the external acceleration. The values of *μ* = 1 and *μ* = 10 were selected to evaluate the effectiveness of the RKF.

**Figure 11:**
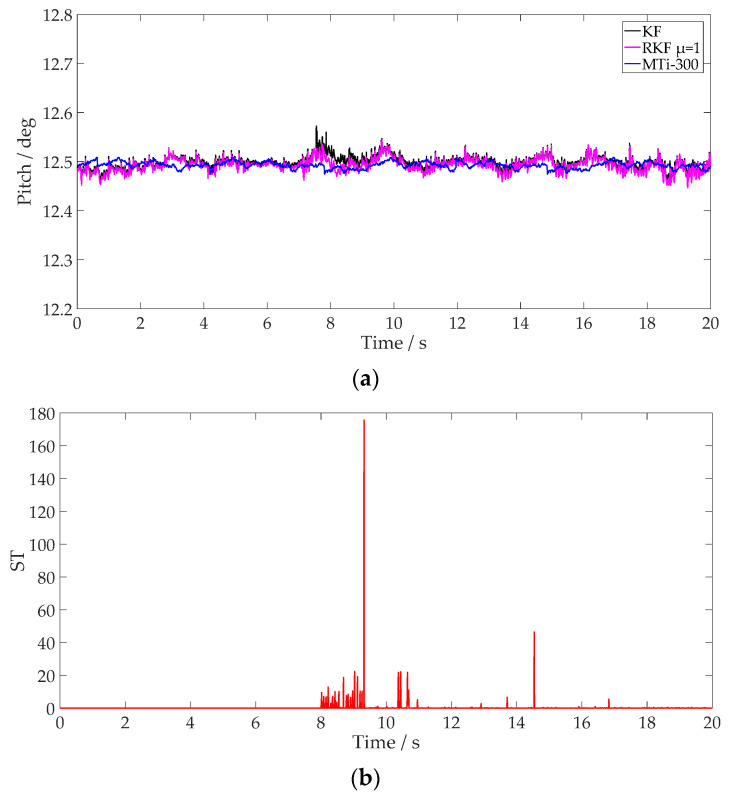
Plough pitch of different methods, using RKF with *μ* = 1. (**a**) Pitch; (**b**) value of ST.

**Figure 12:**
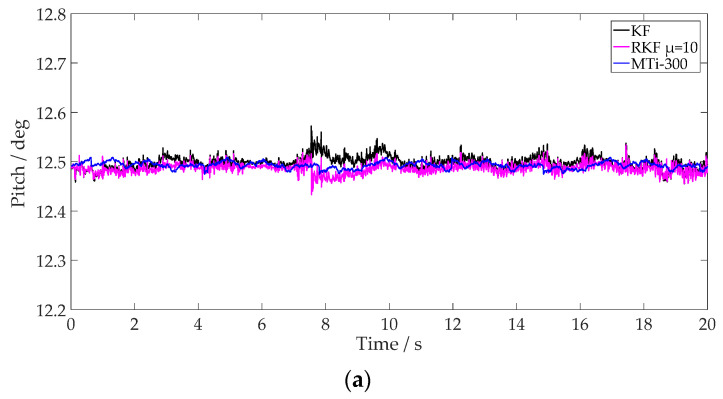

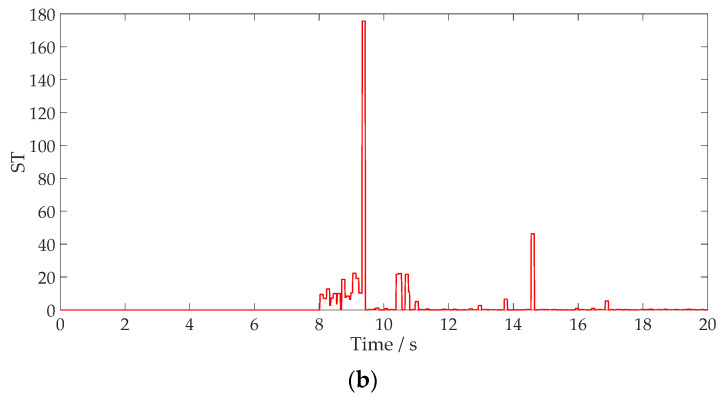
Plough pitch of different methods, using RKF with *μ* = 10. (**a**) Pitch; (**b**) value of ST.

The plough pitch results from different methods and the values of ST for different *μ* settings are shown in Figure 11 and Figure 12. It is notable that the RKF mitigates pitch variations effectively and exhibits a comparable result to that of the MTi-300. Utilizing MTi-300 output as the reference value, Table 3 details the pitch estimations achieved through different methods. Compared with the KF, the RKF demonstrates superior suppression of external acceleration regardless of time window length. The values of ST with a larger time window length are smoother, which can compensate for external acceleration continuously when it occurs during a short period. The pitch result of the RKF with *μ* = 10 is closer to that of the MTi-300 compared with that of the RKF with *μ* = 1. More specifically, the pitch is improved approximately by 47.5% and 31.8%, respectively, compared with that of the KF. The test results indicate that external vibration interference during tractor ploughing operations has a significant effect on the attitude estimation of the plough implement. The RKF proves to be effective in compensating external acceleration interference.

To evaluate the effectiveness of Equation (27) on the detection of external disturbances, this study compares and scrutinizes the attributes of ST value estimation associated with varying values of *μ*. When *μ* is set to 1, ST value exhibits frequent fluctuations, enhancing its ability to differentiate external acceleration changes and increasing its sensitivity. Conversely, when *μ* is set to 10, ST value demonstrates smoother changes, aligning the pitch angle’s variation more closely with the output of the MTi-300. Therefore, ST values under different conditions of *μ* indicate that higher values of *μ* lead to a more accurate estimation of the uncertainty corresponding to unmodeled external acceleration. However, this results in reduced dynamic responsiveness to changes in actual attitude. Given that the adjustment of *μ* necessitates the consideration of carrier attitude response, interference signal characteristics, and algorithmic computation, future research will focus on dynamic window length selection for the RKF for vehicles of different dynamic conditions.

## 4. Conclusions

In order to improve attitude estimation of vehicles operating in complex vibration conditions, this study introduces a multi-scale compensation approach, termed robust KF (RKF), for external acceleration of the MEMS-IMU employed in low-dynamic conditions. The performance of the RKF was validated through experiments based on a three-axis turntable, automatic control vehicle and tractor tillage test, in which its response to different vibration disturbances were analyzed. The results of the turntable experiments demonstrate that the angular tracking error of the RKF outperforms that of the KF and the MTi-300, and the root mean square error (RMSE) is decreased from 0.148° and 0.072°, respectively, to 0.051°. The automatic vehicle and tractor field test demonstrate that the RKF enhances the external acceleration compensation effect significantly in comparison to the KF. Furthermore, the estimation of the external acceleration modeling error is affected by the selected window length for innovation variance estimation. Decreasing the window length can enhance real-time attitude estimation, while increasing it can boost the accuracy of external acceleration compensation and thus can accurately estimate the attitudes of the automatic vehicle and plough.

The experimental results presented in this paper can serve as a valuable reference for the attitude estimation of low-dynamic vehicles, such as monitoring tractor tillage depth based on plough attitude estimation. Future research will focus on exploring adaptive methods for external acceleration modeling, particularly for tillage depth monitoring in the presence of different vibration disturbances.

## Figures and Tables

**Table 1:** Pitch error of different methods.

Methods	Pitch RMSE (°)
KF	0.148
MTi-300	0.072
RKF	0.051

**Table 2:** Attitude error of different methods at different vehicle speeds.

Method	Average Speed (m/s)	Pitch RMSE (°)	Roll RMSE (°)
KF	0.5	0.728	0.965
	1.5	0.875	0.912
RKF	0.5	0.313	0.243
	1.5	0.460	0.495

**Table 3:** Plough pitch error of different filters.

Methods	Pitch RMSE (°)
KF	0.493
RKF *μ* = 1	0.336
RKF *μ* = 10	0.259

## Data Availability

The data presented in this study are available on request from the corresponding author.
